# Efficacy of a “Rescue” Ciprofloxacin-Based Regimen for Eradication of *Helicobacter pylori* Infection after Treatment Failures

**DOI:** 10.1155/2012/484591

**Published:** 2012-05-14

**Authors:** Maria Pina Dore, Vincenza Tadeu, Bianca Are, Ida Mura, Giuseppe Fanciulli, Giovannino Massarelli, Andrea Piana

**Affiliations:** ^1^Clinica Medica, Dipartimento di Medicina Clinica e Sperimentale, Università di Sassari, Viale San Pietro, 8, 07100 Sassari, Italy; ^2^Baylor College of Medicine, Michael E. DeBakey VAMC, 2002 Holcombe Boulevard, Houston, TX 77030, USA; ^3^Sezione di Igiene, Dipartimento di Scienze Biomediche, Università di Sassari, Via Padre Manzella 4, 07100 Sassari, Italy

## Abstract

The aim of our study was to evaluate the efficacy and tolerability of a ciprofloxacin-based regimen for *H. pylori* eradication failures as an alternative to bismuth based quadruple therapy. *Methods*. *Design:* prospective single-center study. Patients in whom a first eradication trial with omeprazole/esomeprazole, clarithromycin plus amoxicillin or tinidazole/metronidazole had failed were included. *H. pylori status:* established by histology, rapide urease test and polymerase chain reaction. *Intervention:* esomeprazole 20 mg, ciprofloxacin 500 mg, and metronidazole 500 mg, administered together before breakfast and dinner for 10 days. Susceptibility testing was performed by the Epsilometer test. Ciprofloxacin resistance was defined as a MIC of ≥1 **μ**g/mL. Eradication was established by a negative 13C-UBT and 4–6 weeks post-therapy. Efficacy and side effects were determined. *Results*. 34 patients were enrolled, 32 completed the study. Compliance was excellent (100%). Side effects were mild. Ciprofloxacin-based therapy cured 65% (22/34) of patients by intention to treat and 69% (22/32) per protocol analysis. The prevalence of ciprofloxacin resistance was 8%. *Conclusions*. The effectiveness of ciprofloxacin-based therapy was greatly reduced despite the high prevalence of ciprofloxacin sensitive *H. pylori* strains. Bismuth based quadruple therapy still remain the best choice as a “rescue” regimen in our region.

## 1. Introduction

*Helicobacter pylori* infection is the main cause of gastroduodenal diseases. Infected individuals develop chronic active gastritis that in a subset of patients might progress to sever complications. The cure of *H. pylori* infection changes the natural history of the gastritis. Antibiotic combination therapy for *H. pylori *eradication is now the standard of care for patients positive for the infection. Guidelines for treatment regimens have been proposed by the European *Helicobacter pylori* Study Group [1].

Proton pump inhibitors (PPIs-)clarithromycin-amoxicillin or metronidazole treatment is the recommended first choice treatment in populations with less than 15 to 20% clarithromycin resistance. In populations with less than 40% metronidazole resistance, PPI-clarithromycin-metronidazole is preferable. Quadruple treatments are alternative first choice treatments. Bismuth-containing quadruple treatments remain the best second choice regimen, if available.

Previous studies demonstrated that in northern Sardinia, 1-week triple therapies consisting of a PPI and metronidazole and clarithromycin or amoxicillin have produced lower than acceptable cure rates, ranging from 55% to 57% [2]. Responsible for most of the reduction in cure rates was the antibiotic resistance to metronidazole, amoxicillin, or clarithromycin. However, resistance to metronidazole can frequently be overcome by increasing the dose and duration of treatment, whereas clarithromycin resistance cannot [3]. Colloidal bismuth subcitrate-based twice-a-day quadruple therapy demonstrated to be an excellent primary therapy with an intention-to-treat (ITT) cure rate of 95% (95%; CI: 90–98%) and 98% per protocol (PP), irrespective of diagnosis, age, prior treatment failure, or smoking status [4]. In addition the colloidal bismuth subcitrate-based twice-a-day quadruple regimen showed a high efficacy for patients failing one or more courses of *H. pylori* treatment in Sardinia [5].

Other combinations using different medications have been proposed as “rescue” therapies including therapy with fluoroquinolones [6–9] with a PPI [10], reserved in general, for patients who fail two courses of treatment. However, in few studies the efficacy of ciprofloxacin-based regimen was explored [11–15].

In order to offer an alternative to our patients with *H. pylori* infection after a treatment failure we evaluated the efficacy and tolerability of a Ciprofloxacin-based regimen.

## 2. Methods

### 2.1. Patients

 This study was a prospective, single center trial for treatment of consecutive patients in whom a first eradication trial with omeprazole/esomeprazole, clarithromycin, and amoxicillin (14 patients) or omeprazole/esomeprazole, clarithromycin, and tinidazole/metronidazole (20 patients), had failed to eradicate *H. pylori *infection.

### 2.2. Exclusion Criteria

 Patients who received bismuth compounds, antisecretory drugs, or antibiotics during the 4 weeks before endoscopy were excluded. Other exclusion criteria included pregnancy or lactation; regular use of nonsteroidal anti-inflammatory drugs including acetylsalicylic acid and/or corticosteroids, malignancy, severe liver, heart, kidney or endocrine diseases, and any other clinically significant medical condition, alcohol abuse, drug addiction, history of allergy to any of the drug used in the study. Written informed consent was obtained from all participants, and the study was conducted according to the guidelines of the local ethics committee.

### 2.3. Definition of *H. pylori* Infection

 Pretreatment *H. pylori* infection was defined as a positive rapid urease test (CP-test, Yamanouchi S.p.A., Milan, Italy), the presence of *H. pylori* on histological examination, and by polymerase chain reaction (PCR) analysis. Posttreatment success was defined by a negative 13C-UBT 4-5 weeks after completing therapy.

### 2.4. Histology

 Two biopsy specimens were taken from the *antrum*, one from the *angulus* and one from the gastric corpus for histology. Biopsy specimens were immediately fixed in 10% buffered formalin and subsequently stained with hematoxylin and eosin and with Giemsa to assess the presence of *H. pylori.*

### 2.5. 13C-Urea Breath Test

 The 13C-UBT was performed according to a standardized protocol, the sensitivity, and specificity of which have been reported to be of 95% [16]. All breath tests were analyzed at the same laboratory by using a single gas isotope ratio mass spectrometer (ABCA, Europe Scientific, Crewe, UK).

### 2.6. Microbiology

One antral biopsy was immediately transported at room temperature in a Portagerm pylori (bioMerieux, S.p.A., Rome, Italy) for culture. Biopsies were streaked on Columbia agar plates (bioMerieux) and were incubated for 3–5 days at 37°C, 12% CO_2_ (Campy Pak Plus, BBL Becton Dickinson, Cockesville, MD) and 100% relative humidity. The identity of *H. pylori* was confirmed by colony appearance, Gram stain, and positive biochemical tests (oxidase, catalase, and urease). The minimum inhibitory concentration (MIC) was determined by the *E*-test (AB Biodisk, Uppsala, Sweden) following the manufacturer's instructions in Columbia agar plates (90 mm) containing 5% sheep blood as previously described [2]. *E-*test strips were aseptically placed onto dried surfaces of the inoculated plates. Test results were read after incubation at 37°C for 72 h in a standard atmosphere. MICs were determined by the intercept of the elliptical zone of inhibition with the graded *E*-test strip. The breakpoints used to define resistant *H. pylori* were for amoxicillin ≥8 *μ*g/mL, clarithromycin ≥2 *μ*g/mL, metronidazole ≥2 *μ*g/mL, and for tetracycline ≥2 *μ*g/mL, respectively [2].

The cutoff concentrations used to define ciprofloxacin resistance was ≥1 *μ*g/mL [17, 18]. Post-treatment culture of biopsy specimens and antibiotic susceptibility tests were not done.

### 2.7. Genomic Characterization of *H. pylori* by Polymerase Chain Reaction

 Genomic DNA was extracted from gastric biopsies collected one from the *antrum* and one from the *corpus* using the QIAamp Tissue kit (QIAGEN, Inc., Chatsworth, CA, USA), according to the manufacturer's instructions.

A set of primers specific for the *H. pylori vacA *gene (VAG-F: 5′-CAA TCT GTC CAA TCA AGC GAG-3′ and VAG-R: 5′ GCG TCA AAA TAA TTC CAA GG-3′) with a reported sensitivity of 98.4% [19] was used to amplify a 570-bp product for *m1 *and 645-bp product for *m2 *from the middle region of the *vacA *gene [20].

### 2.8. Medication

The intervention consisted of esomeprazole 20 mg, plus ciprofloxacin 500 mg, and metronidazole 500 mg bid. The choice of ciprofloxacin was based on the fact that the efficacy of ciprofloxacin-based regimen was evaluated in a small number of studies [11–15] and never in Sardinia. No treatment was administered thereafter. All drugs were administered together before breakfast and dinner for 10 days. The duration of 10 days was arbitrarily decided.

### 2.9. Patient Compliance

 The patients were evaluated for compliance at followup, after completing treatment, by a physician, both by direct questioning and by pill count and were scored according to the number of days the patient took the study medication. All patients were interviewed for possible side effects by questionnaire. Side effects were graded as mild (did not limit daily activities), moderate (limited daily activities to some extent), or severe (made daily activities all but impossible).

### 2.10. Statistical Analysis

 For quantitative variables, percentage and 95% confidence interval (CI) were calculated. Analysis of *H. pylori* eradication efficacy was performed on an “intention-to-treat” (ITT) basis including all eligible patients enrolled in the study and on a “per-protocol” (PP) basis excluding patients lost to the followup.

## 3. Results

A total of 34 consecutive patients fulfilling the inclusion criteria were enrolled in the study (12 males, median age 55,6 years range 25–75 years).

No one patient had peptic ulcer disease, gastric, nor duodenal erosions. All patients had experienced dyspeptic symptoms. Thirty-two patients took medications as prescribed and* H. pylori *eradication status was assessed after therapy. Two patients were lost to followup.

### 3.1. *H. pylori* Eradication

 Ciprofloxacin-based therapy cured 65% (22/34; 95% CI: 46–80) of patients with *H. pylori* infection by ITT analysis and 69% (22/32; 95% CI: 50–83) by PP analysis (Table 1). Patients harboring ciprofloxacin-resistant *H. pylori* strains were not cured. Treatment failures by ciprofloxacin-based “rescue” therapy were cured with bismuth-based quadruple therapy bid.

### 3.2. Patient Compliance and Side Effects

Overall tolerability was good. Excellent compliance (100%) was achieved in all cases. No one pill was returned by the patients. Data on side effects were available for all 32 treated patients. No one patient complained severe side effects. Fourteen patients (9 women) developed mild side effects such as fatigue, diarrhea, nausea, vomiting, or stomachache, making working more difficult in the final treatment days.

### 3.3. Antibiotic *H. pylori* Resistance

Pretreatment culture was available for 26 patients. Susceptibility testing was not possible in 6 cases because of absence of microbial growth due to insufficient gastric tissue. Two patients (8%) harbored *H. pylori *resistant to ciprofloxacin, and amoxicillin, one *H. pylori* isolate (4%) was resistant to clarithromycin and 3 (12%) to metronidazole. All strains were susceptible to tetracycline (Table 2).

### 3.4. Genomic Characterization of *H. pylori Organisms*

 DNA analysis by PCR amplified the middle region of *vacA *gene in all gastric samples. In 29 cases the genomic fragment of *vacA m2* subtype, more prevalent in our region [20, 21], was amplified and in 6 cases *vacA m1 *subtype.

## 4. Discussion

Despite the large number of studies, identifing an optimal therapeutic regimen for *H. pylori* infection remains a challenge. Quadruple therapy consisting of a PPI combined with bismuth (525 mg four times daily) and two antibiotics (e.g., metronidazole 250 mg four times daily and tetracycline 500 mg four times daily) given for 10 to 14 days has been generally used as the optimal second-line therapy after proton pump inhibitor clarithromycin amoxicillin failure and has been the recommended rescue regimen in several guidelines. The introduction of combination capsules containing bismuth-quadruple therapy has led to a resurgence in interest in bismuth-containing regimens as appropriate first-line therapy [22–24].

Quadruple therapy is especially appropriate as initial therapy in areas in which the prevalence of resistance to clarithromycin or metronidazole is greater than 20% or in patients with recent or repeated exposure to clarithromycin or metronidazole for any reason [25]. We previously showed that a modified schedule in which quadruple therapy was given twice-a-day for 14 days (i.e., with the noon and evening meals) yielded high cure rates [4], despite the fact that our geographic area has a high background prevalence of antibiotic resistance [2]. For example the reported prevalence of antibiotic resistance in Sardinia was 29% for metronidazole, 26% for amoxicillin, 23% for clarithromycin, 14% for tetracycline, and 33% for doxycycline [2]. In addition midday bismuth-subcitrate-based twice-a-day quadruple therapy demonstrated to be excellent also as a “rescue” therapy. The reported cure rates were for intention to treat = 93% (95% CI = 84% to 98%) and per protocol = 97% (95% CI = 89% to 100%) [5].

In order to offer an alternative to quadruple regimen to our patients we evaluated the efficacy and tolerability of a ciprofloxacin-based regimen in patients with *H. pylori* infection after a treatment failure.

Ciprofloxacin belongs to the fluoroquinolone group of antibiotics that are generally used as part of rescue therapy for treating *H. pylori* infections when first- and second-line therapies have failed [26, 27]. Resistance to fluoroquinolones is generally very low (<10%) worldwide [28]. In addition ciprofloxacin has low MICs against *H. pylori* and is highly active *in vitro* in eradicating the organism [29], and high levels of ciprofloxacin have been found in gastric tissue biopsy specimens [30].

In small early trials ciprofloxacin failed to show efficacy against *H. pylori* because of a reduced antibiotic activity in an acid pH environment due to increasing ionization [31, 32]. Based on this information, in further trials, in order to enhance *in vivo* activity, ciprofloxacin was administered with acid-suppressing drug such as omeprazole and cure rates of 70% were obtained [9]. Similar results (70%) were confirmed in Europe given ciprofloxacin as a component of a sequential therapy for patients with metronidazole-resistant strains [12]. In Iran a regimen consisting of omeprazole 20 mg plus bismuth subcitrate 120 mg 2 tab. plus ciprofloxacin 500 mg bid achieved an eradication rate of 67.1% [14]. In the same country similar results (65,4% and 70% by ITT and PP analyses) were obtained given omeprazole and amoxicillin, both twice daily for two weeks and ciprofloxacin twice a day for the first week [15].

In our study, ciprofloxacin-based therapy cured 65% (22/34) of patients with *H. pylori* infection by ITT analysis and 69% (22/32) per PP analysis beside the low rate of ciprofloxacin resistance among strains (2 out 26).

There are no data about resistance of *H. pylori* to ciprofloxacin in our country; however, primary resistance to levofloxacin in Italy has been demonstrated to be low [33]. The likelihood of treatment eradication success can be improved through the use of antibiotics that are known to be most effective in the region. According to this notion reported eradication rates with levofloxacin-250 sequential therapy and with levofloxacin-500 sequential therapy were 97% and 96% by the ITT analyses in Italy [34].

In the other hand Güzelbulut et al. have shown that in Turkey, a modified sequential therapy regimen, consisting of PPI plus amoxicillin (5 days) followed by PPI plus levofloxacin (5 days) was not superior (ITT: 67%) over standard sequential therapy for *H. pylori* eradication [35].

We do not have a convincing explanation about the numerous factors that could have affected eradication rates in our study. We may suppose that the drug formulation used, chemical properties of the drug, its stability at varying pH, concentrations of active drug in the gastric tissue, frequency, and timing of drug or the regimen combination could have interfered in the final efficacy of ciprofloxacin-based treatment.

In conclusion, based on the results of our study we have to consider ciprofloxacin-based treatment a nonacceptable option as a “rescue” treatment in our region.

## Figures and Tables

**Table 1:** Intervention status of patients enrolled in the study.

Status	Patients*
Received intervention	34
Lost to followup	2
Completed trial	32
Intervention ineffective	10
Cure Rate ITT	65% (22/34)
95% CI	46–80
Cure Rate PP	69% (22/32)
95% CI	50–83

*Patients in whom a first eradication trial with omeprazole/esomeprazole, clarithromycin, and amoxicillin (14 patients) or omeprazole/esomeprazole, clarithromycin, and tinidazole/metronidazole (20 patients) had failed to eradicate *H. pylori* infection.

ITT: intention-to-treat analysis; PP: per protocol analysis; CI: Confidence Interval.

**Table 2:** Prevalence of antibiotic resistance of *H. pylori* isolates after treatment with omeprazole/esomeprazole, clarithromycin, and amoxicillin or omeprazole/esomeprazole, clarithromycin, and tinidazole/metronidazole.

Antibiotics	MIC cut off (number of *H. pylori-*resistant strains)
Amoxicillin	>8 *μ*g/mL (2 > 8 *μ*g/mL)
Clarithromycin	≥2 *μ*g/mL (1 > 256 *μ*g/mL)
Metronidazole	>8 *μ*g/mL (3 > 256 *μ*g/mL)
Ciprofloxacin	≥1 *μ*g/mL (2 > 32 *μ*g/mL)
Tetracycline	≥2 *μ*g/mL (0)

MIC: minimum inhibitory concentration.
